# Examining modifications of execution strategies during a continuous task

**DOI:** 10.1038/s41598-021-84369-5

**Published:** 2021-03-01

**Authors:** Erez James Cohen, Kunlin Wei, Diego Minciacchi

**Affiliations:** 1 Department of Experimental and Clinical Medicine, Physiological Sciences Section, University of Florence, Viale Morgagni 63, 50134 Florence, Italy; 2 School of Psychological and Cognitive Sciences and Beijing Key Laboratory of Behavior and Mental Health, Peking University, Beijing, China

**Keywords:** Neuroscience, Motor control

## Abstract

How strategies are formulated during a performance is an important aspect of motor control. Knowledge of the strategy employed in a task may help subjects achieve better performances, as it would help to evidence other possible strategies that could be used as well as help perfect a certain strategy. We sought to investigate how much of a performance is conditioned by the initial state and whether behavior throughout the performance is modified within a short timescale. In other words, we focus on the process of execution and not on the outcome. To this scope we used a repeated continuous circle tracing task. Performances were decomposed into different components (i.e., execution variables) whose combination is able to numerically determine movement outcome. By identifying execution variables of speed and duration, we created an execution space and a solution manifold (i.e., combinations of execution variables yielding zero discrepancy from the desired outcome) and divided the subjects according to their initial performance in that space into speed preference, duration preference, and no-preference groups. We demonstrated that specific strategies may be identified in a continuous task, and strategies remain relatively stable throughout the performance. Moreover, as performances remained stable, the initial location in the execution space can be used to determine the subject’s strategy. Finally, contrary to other studies, we demonstrated that, in a continuous task, performances were associated with reduced exploration of the execution space.

## Introduction

When we consider a redundant goal directed task as a motor problem, the ways to solve the problem are considered as the possible strategies for said task. The number of possible strategies in a task may be immense and is dependent on various factors. It was shown that factors such as motor variability^1–3^, working memory^4^, visual processing^5^, visual-motor connectivity^6^, to previous experience^7^, age^8^, as well as subjects’ specific preference of a strategy^9,10^ could influence performances, all of which may not be predictable a priori. In fact, it was argued that observed patterns of execution are more emergent rather than prescribed properties and, as such, are dependent on various constraints which were summarized as environmental-, organism-, and task-related^11^. Therefore, it is difficult to determine what strategy may be employed by subjects when left to their own devices as it is not feasible to account for all of these factors. Still, depending on what is being examined, there may be a way to overcome this.

If a performance is considered as an exploration (i.e., a systematic pattern over time that emerges from the interaction between individual and environment in pursuit of the task goal)^12^, the exploration in itself could represent a quantifiable measure, independent of outcome, which could provide additional information regarding the performance. In fact, several studies have investigated evolution of performances in terms of exploration by using decomposition methods^13–15^. These decomposition methods all share a common feature, that of examining certain parameters during movement and mapping the relationships of said parameters relative to performance outcome. Such methods have been used for classification^13^, solutions to (cope with) redundancy^14^, identification of task relevant and irrelevant variability^15^, and success in relation to variability^16^. To our knowledge these techniques were not fully implemented to examine modifications of strategies during a performance based on the initial conditions.

It is worth mentioning that King and colleagues, 2012^9^, by examining the interplay of execution variables, demonstrated that specific strategies can be identified by the initial performance. Furthermore, the study reported high exploration across subjects. However, the study used a discrete approach which may favor exploration. In fact, it was shown that when reducing inter-trial interval and pre-movement planning, people tend to rely less on a specific strategy but more on error based modifications^17,18^. As such, in discrete tasks, as more time is present to elaborate previous performance, subsequent performances may vary greatly. In continuous tasks on the other hand, pre-movement planning is less important, consequently there is an ongoing regulation of control^19,20^. Hence, when it comes to exploration patterns, it is possible that in a continuous task subjects would only make incremental changes, as tasks are to be performed smoothly. Consequently, abrupt changes in performance (e.g., following an unsuccessful trial) are less likely to occur, which could provide different results compared to those reported by King et al., 2012.

With these considerations in mind, in the present study a circle tracing task was asked to be performed as fast and accurate as possible. Given the continuous nature of the task and the short time span of execution, we hypothesize that no abrupt changes in behavior would occur. Therefore, specific execution strategies could be identified by the initial state and remain relatively fixed throughout the performance. Furthermore, we hypothesize that exploration would be greatly reduced. Moreover, similarly to King and Colleagues, we hypothesize that subjects could be classified into distinct groups, based on their execution, with distinct characteristics.

## Material and methods

### Participants

40 healthy adults were recruited for this study (age: 20.27 ± 2.93 years; 17 males). All participants were right-handed. Participants were naive to the task and the purpose of the study, and free of documented neurological impairments. All participants reported to have a corrected-to-normal visual acuity. The study protocol was approved by the Institutional Review Board of Peking University and all procedures conformed to the code of ethics of the Declaration of Helsinki. All participants gave written informed consent and were paid for their time.

### Set up and task

**Figure 1:**
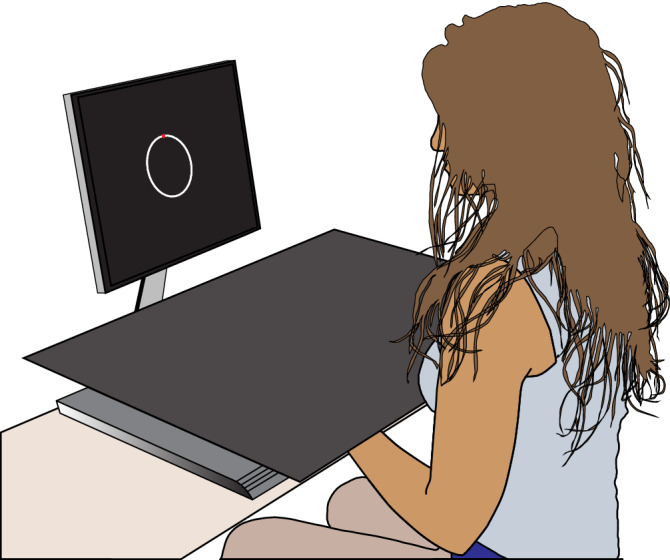
Setup. Diagram illustrating the experimental setup. Each subject was presented a circle template projected on a monitor in front of her/him at eye level. A black paperboard occluded vision of the hand. The subjects executed tracings of a circle, while seated without the support of either wrist, arm, or elbow, in such a way that the only contact with the tablet was made through the pen.

The setup is similar to the one used in Cohen et al.^20^, and is briefly summarized here. Participants were presented a circle template projected on a monitor mounted vertically in front of them at eye level (Fig. 1). A black paperboard occluded vision of the hand in order to facilitate fixation on the screen. On the circle template (2.27 cm in radius) a small moveable red dot represented the starting point (set to 12 o’clock). The participants were instructed to execute tracings of a circle, using graphic pen tablet (Wacom Intuos PTK-1240, Tokyo, Japan; active area: 462 × 305 mm; sample rate 60 Hz), while seated without the support of either wrist, arm, or elbow, in such a way that the only contact with the tablet was made through the pen. Further instructions included tracing the target circle counterclockwise as fast and as accurate as possible. Before starting the task, each participant was asked whether the instructions were understood. Once participants positioned themselves at the correct point, the small dot turned to green indicating the start of the trial and the cursor became invisible. During execution, the cursor position, represented by the small dot, was visible. The cursor trajectory was also visible, and was reset every revolution (defined as return to 12 o’clock that occurs in a direction from right to left, i.e., anticlockwise). Each participant was tested individually and was asked to continuously trace the circle without stopping. Once 48 revolutions of the circle were traced, the trial ended automatically. On average, the duration of the trial lasted 42.3 ± 12.16 s.

### Analysis

#### Circle analysis

Circle tracing analysis consisted of calculation of traced circle radii, measured as point distances from the template’s center (i.e., radius). To avoid over estimations, the tracing of the circle was first reduced to 360 points (1 point per angle). Following that, all other calculations were performed. Specifically, for each point drawn, the distance from said point to the template center was calculated and was considered as a measure for the radius for said point. In order to obtain a radius value for each revolution, the mean value of the radii measured for each revolution was considered along with the standard deviation for said revolution. For each measured radius, deviations from the template’s radius were also calculated (i.e., radius error) which were used to calculate the mean radius error for each revolution. Since we are interested in overall error reduction, the radius error values were considered as absolute values. The sum of the radius errors for each revolution was considered as the total error. In addition, for each revolution the travelled distance was calculated. This measure was further used along with the duration of each revolution to estimate the speed for said revolution (derived from Speed = Distance / Duration). Finally, for evaluation of inter-revolution differences, the differences between two successive revolutions were calculated, for both duration and speed.

#### Speed profile

The number of peaks within the speed profile of the entire performance of each subject was used as an estimate for movement smoothness^21,22^. Speed data of the performances was first filtered using local regression with weighted linear least squares and a 1st degree polynomial model setting, span was set for 1% of the dataset’s length. Following that using Matlab R2019b, the find peaks algorithm was implemented in order to calculate the number of peaks.

#### Timing variability

In order to evaluate timing variability within the performances, the detrended windowed lag-one autocorrelation (detrended-wγ(1)) was used on the measured duration of each revolution for each subject^23^. This type of analysis further allows for a separation of two types of timing control mechanisms: event-based, which assumes that timing is controlled according to an internal representation^24,25^, and would therefore be characterized by negative detrended-wγ(1); and emergent-based, which considers that timing control emerges from performance dynamics rather than being actively compared with an internal representation^26^, and is characterized by positive detrended-wγ(1). We computed wγ(1) over a window of the 30 first points, moving the window by one point, all along the sets. For each point moved the data was first linearly detrended on the window, and then the lag-one autocorrelation was computed. Following that the mean detrended-wγ(1) was calculated for each subject, and was considered as an estimator of the overall autocorrelation.

#### Creation of execution space and solution manifold

**Figure 2:**
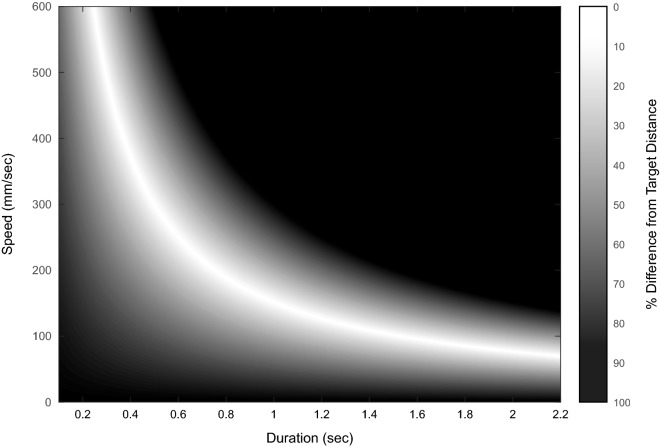
Execution space. Within the possible combination of the execution variables (duration and speed), the most congruent combinations with the target distance create the solution manifold (represented in white). The better the combination of the variables is (i.e., the closer their combination yields the target distance) the whiter the area is.

In order to examine exploration patterns within the data, an execution space (i.e., a numerical representation of all possible execution patterns based on specific performance variables) is needed to be created. To achieve this, first a result variable is to be chosen. This variable must represent some measure that could be compared with the desired outcome. In the circle tracing task, since the distance travelled each revolution could be readily compared with the target distance (i.e., circumference of the template circle), it was chosen as the result variable. Following this, two execution variables are to be chosen. The relationship of these execution variables must be able to quantify the result variable. When examining the measurable quantities retrieved in this task (i.e., trajectory and time), as well as the result variable, it seems fitting to define the execution variables from the distance and time relationship (i.e., Distance = Speed × Duration). Consequently, since the distance measure is considered as the result variable, we may use Speed and Duration as the execution variables. This allows for the creation of an execution space based on the various combinations of speed and duration, and a solution manifold for the combinations yielding zero discrepancy between the circumference of the target circle and the given combination^28^ (Fig. 2).

The execution space was created by pairing the possible values of each of the execution variables. The limits of the space were chosen a posteriori following the examination of the dataset and were set to maximum values of 2.2 s for duration, and 600 mm/s for speed. Following that, the distances calculated using the various combinations of speed and duration were mapped. For visualization purposes, we have arbitrarily chosen a 600 × 600 matrix of distances calculated using the various combinations of speed and duration (0–2.2 s for duration; 0–600 mm/s for speed). Each value obtained was thereafter compared with the template’s circumference (measured 142.85 mm) using the formula: (calculated distance)/(template’s circumference) to yield a ratio which later is used as a measure of the discrepancy between the given combination and the circumference of the template (i.e., solution manifold; Fig. 2). We considered 0 as no discrepancy between the calculated distance and the template’s circumference. The closer the value was to 0, the whiter the area corresponding to the combination of variables.

#### Cluster analysis and data division

A k-means algorithm was used in order to divide subjects into groups based on their initial performance, specifically based on speed and duration values. The reason for choosing k-means clustering was that it does not require any prior assumption regarding the performance. In order to determine how many clusters should be used for k-means clustering, we have conducted a Cluster Solutions Evaluation analysis using the silhouette criterion. The silhouette value is a measure of how similar an object is to its own cluster (compactness) compared to other clusters (separation)^27^. We have examined division of the data up to 10 clusters.

#### Tolerance, noise and covariance-cost analysis

To examine determinants of performance the Tolerance, Noise and Covariance—Cost (TNC-Cost) analysis^28^ was implemented. The analysis method is fully described in Cohen and Sternad, 2009, and is briefly summarized here. According TNC-Cost analysis, Tolerance Cost is considered to quantify the cost (measured as distance) related to an insufficient exploration of the solution space. Noise Cost is considered to measure the cost for a less than optimal dispersion. Finally, Covariance Cost quantifies the cost of not sufficiently exploiting the redundancy of the already explored space.

**Figure 3:**
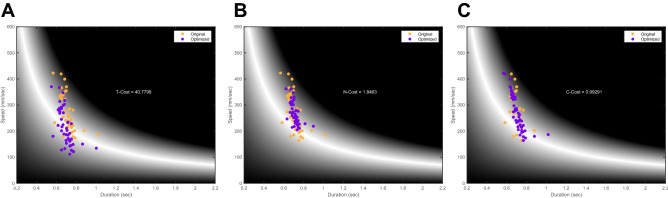
TNC-cost. Example for the calculation of the TNC-Cost for taken from a single subject throughout the performance. Each dot represents a single revolution performed; colors correspond to the original data (orange) compared to the optimized data (purple). (**A**) It is possible to see that for T-Cost, the entire data set was shifted to the location within the execution space that provided the best mean solution, while maintaining dispersion of data. (**B**) For the N-Cost, the data remained in the same location but the dispersion was minimized to the point which provided the best overall solution. (**C**) C-cost was calculated as the best possible combination of the actual performed values compared the original combinations.

To evaluate Tolerance Cost (T-Cost), an optimized set of data was generated in which the mean of the speeds and the mean of the durations were shifted to location that provided the best overall result, while still maintaining the dispersion of original data along the axes. To achieve this, first a grid of 600 × 600 was created on which the dataset was shifted. For each shift the difference between the mean distance (product of the execution variables) and the circumference were compared. The location that yielded the best overall performance result was compared with the result of the actual dataset to define the T-Cost (Fig. 3A).

For Noise Cost (N-Cost), the radial distances between each point and the mean value of the dataset were calculated. Following that, radial distances were divided to 100 steps, each step was used to “shrink” the data closer to its mean until finally all points collapsed to the mean duration and speed at the 100th step. At each step the mean distance was calculated and compared with the circumference. The step that yielded the best overall result was considered as the optimal noise for the performance and was compared with the mean distance of the original dataset, defining the N-Cost (Fig. 3B).

Covariance Cost (C-Cost) was evaluated by first ranking pairs of durations and speeds from best to worst. Then systematically swapping the speed values in a manner that first worst duration was paired with second worst speed (and vice versa). If the mean result improved over the original, the swap was accepted. This was continued until the worst duration was paired with the best speed. The same process was repeated for duration. The number of profitable swaps was recorded and the process repeated until no further profitable swaps could be made (optimal permutation of the data was obtained). The algebraic difference between the mean distance of the actual data set and the mean distance of the optimally recombined set defined C-Cost (Fig. 3C).

### Statistics

To evaluate the suitability of distance as the result variable, Pearson correlation with Bonferroni correction was implemented on the distance measured per revolution and the average radius measured per revolution, across all subjects and revolutions.

To evaluate the improvement of the performance, paired sample t-tests were implemented on the initial error and end error (i.e., average of the first and last revolutions, respectively, across subjects) for both radius error as well as for total error.

A non-parametric analysis was conducted due to the small number of participants following group division and the fact that 1 (speed) out of 3 (radius, duration, and speed) main variables was not normally distributed (Shapiro–Wilk test, p < 0.001). For comparisons between groups, the Kruskal–Wallis test was used followed by a Dunn-Bonferroni adjusted post hoc test in case of multiple comparisons. The analyses were conducted specifically on inter-revolution differences for speed and duration, as well as on the peak numbers in the speed profiles and on the detrended-wγ(1) values. Unless stated otherwise, the values reported in the study are considered as mean and standard deviation. All of the statistical analyses performed in this study were done using Matlab R2019b.

## Results

**Figure 4:**
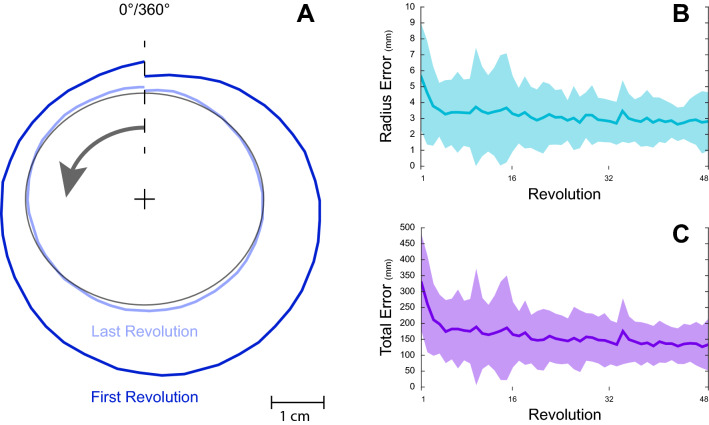
Example traces and error reduction. (**A**) Example of traces performed by a subject. The black circle represents the template on which tracings were performed in a counterclockwise manner (arrow). In dark blue is the tracing of the first revolution, whereas in light blue is the tracing of the last revolution. (**B**,**C**) Results for radius error (**B**) and for total error (**C**) per revolution for all subjects, it is possible to see that both radius and total errors gradually reduce during the task. Specifically, initial radius error reduced from 5.65 ± 3.35 mm for the first revolution to 2.81 ± 1.83 mm by the last revolution, and total error reduced from 329.2 ± 151.7 mm to 133.47 ± 81 mm.

As a first measure, we evaluated the task execution according to the instructions. We assume that if the instructions were indeed followed, there should be a gradual reduction in error (Fig. 4). In fact, the initial radius error for the first revolution measured 5.65 ± 3.35 mm, reducing to 2.81 ± 1.83 mm (t(39) = 4.402, p < 0.001) by the last revolution (Fig. 4B), total error reduced from 329.2 ± 151.7 mm to 133.47 ± 81 mm (t(39) = 6.578, p < 0.001) (Fig. 4C).

To evaluate the suitability of distance as the result variable, which is a pre-requisite for the creation of the solution manifold, the Pearson correlation coefficient, along with a Bonferroni correction (considering a sample size of 40), was used and revealed a significant correlation between distance and radius across all subjects and revolutions. This was done on a subject level so as to assure the suitability of distance as the result variable for all subjects, regardless of their patterns of execution. Specifically, all r-values were found to be larger than 0.9 with p-values < 0.001. The average correlation measured 0.97 ± 0.016 across all subjects and revolutions (Table 1). Therefore, since the radius error derives from the radius measurement, it is safe to say that the distance measure, by having a very high correlation with the radius, may represent a suitable result variable.

**Table 1:** Radius and distance correlation results.

Subject	r-value
1	0.97
2	0.98
3	0.97
4	0.98
5	0.98
6	0.95
7	0.97
8	0.98
9	0.98
10	0.96
11	0.97
12	0.97
13	0.97
14	0.96
15	0.95
16	0.97
17	0.96
18	0.99
19	0.96
20	0.94
21	0.94
22	0.97
23	0.93
24	0.98
25	0.98
26	0.96
27	0.98
28	0.98
29	0.97
30	0.97
31	0.90
32	0.98
33	0.98
34	0.97
35	0.97
36	0.97
37	0.97
38	0.98
39	0.97
40	0.98

In the table, the left column refers to the subject, whereas the right column reports the correlation results between the radius and distance measured across all revolutions.

The solution manifold within the execution space assumes curved conformation. Furthermore, it is possible to see that the solution manifold becomes larger toward the center (i.e., larger area in light gray shade) of the curve and narrower at the extremes (see Fig. 2). This larger area is considered to be less prone to error or to possess a greater tolerance, meaning that performances within that area are less sensitive to small variations of both execution variables. It was demonstrated that as performances progress there is a general tendency toward this area of greater tolerance as it allows for the best exchange between the variables^28^. Therefore, we can assume that if performances do indeed respect the architecture of the solution manifold, there should be a tendency to converge toward the area of greater tolerance.

**Figure 5:**
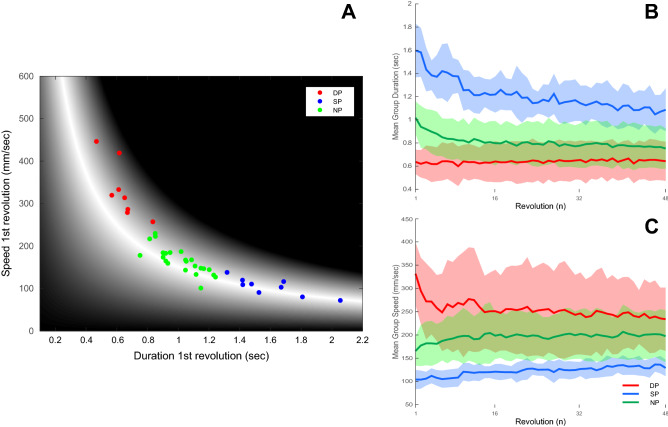
Group division. Subjects were subdivided into 3 groups following the implementation of k-means on the speed and duration values of the first revolution. (**A**) Scatter plot of the speed and duration values obtained on the first revolution, every dot represents a subject and is colored according to the k-means division into a Duration Preference group (i.e., DP; n = 8; red), Speed Preference group (i.e., SP; n = 9; blue) and No Preference Group (i.e., NP; n = 23; green). Arrows represent the quiver plot obtained by taking the mean of the first revolution and the last revolution for each group. (**B**) Duration results throughout the performance for the 3 groups. It is possible to note that the DP group (red) remains relatively stable compared to the SP group (blue). Specifically, the DP group mean duration measured 0.63 ± 0.104 s for the first revolution, and remained relatively stable throughout the performance reaching 0.64 ± 0.168 s by the last revolution. The SP group measured 1.59 ± 0.23 s for the first revolution, reaching 1.08 ± 0.18 s by the last revolution, and the NP group measured 1.01 ± 0.14 s for the first revolution, reaching 0.75 ± 0.174 s by the last revolution. (**C**) Speed results throughout the performance for the 3 groups, demonstrating that SP group (blue) remains relatively stable compared to the DP group (red). Specifically, the DP group averaged 331.6 ± 67.2 mm/s for the first revolution, reaching 233.8 ± 68.05 for the last revolution. The SP group averaged 104.2 ± 20.5 mm/s for the first revolution and remained relatively stable measuring 129.1 ± 17.7 mm/s by the last revolution. The NP group averaged 165.5 ± 31.1 mm/s for the first revolution, increasing to 197.4 ± 55.9 mm/s by the last revolution.

It appears that the solution manifold could be divided into two parts relative to the area of greater tolerance, one part would correspond to relatively small changes in speed with large changes in duration, and the second part with the opposite trend. From this, two strategies emerge, the first is that of maintaining a relatively constant speed, the other of maintaining a relatively constant duration. Therefore, the initial location of the subject within the execution space, would very likely determine said subject’s exploration strategy. We should however also consider the possibility that certain subjects, specifically those around the area of greater tolerance in the solution manifold, may present intermediate values, similarly to what was found by King and colleagues^9^. Therefore, in order to avoid assumptions, we have conducted a Cluster Solutions Evaluation analysis using Matlab, which revealed that 3 clusters are the most appropriate for the data in this study (criterion value = 0.73). This a priori knowledge was used to divide the subjects into 3 groups using a k-means algorithm, using only the speed and duration of the first revolution (results for 2 groups division are included in the Supplementary Material S1 of this manuscript). The 3 groups obtained were that of steady speed (i.e., Speed Preference, SP) and steady duration (i.e., Duration Preference, DP) and No-Preference (NP) group (SP; n = 9, DP; n = 8, NP; n = 23; Fig. 5A). Particularly, the average duration measured 0.63 ± 0.01 s for the DP group, 1.2 ± 0.12 s for the SP group and 0.8 ± 0.04 s for the NP group. The average speed measured 254 ± 16 mm/sec for the DP group, 122 ± 9.1 for the SP group and 195 ± 7.6 for the NP group. Both duration and speed were found to be significantly different among groups (duration: Chi sq(2) = 127.1, p < 0.001; speed: Chi sq(3) = 127.1, p < 0.001). Post hoc analyses also confirmed significance between all groups (duration p < 0.001 for DP vs SP, SP vs NP, DP vs NP; speed p < 0.001 for DP vs SP, SP vs NP, DP vs NP).

**Figure 6:**
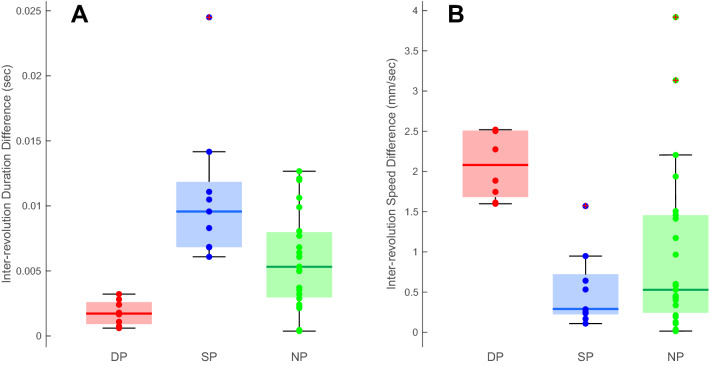
Inter-revolution differences. Box plot representing the group inter-revolution differences for duration (**A**) and for speed (**B**). It is possible to see that, for duration, DP group (red) presents very small differences compared to SP (blue), suggesting that DP group maintains the duration relatively constant. The opposite trend is visible for speed in which the SP group (blue) presents smaller differences compared to the DP group (red). The NP groups presented intermediate values for both duration and speed (green). The colored dots superimposed on the plots represent the individual subject and are color coded according to their group (red for DP group, blue for SP group, and green for NP group). Crosses represent extreme outliers.

If strategies are indeed fixed, there should be a minimal change in duration for the DP group along with large changes in duration for the SP group (Fig. 5B). The opposite should be visible when examining the speed parameter (Fig. 5C). Consequently, performances would be accommodated by a greater modification of a single execution variable. Since using the speed and duration values would evidently be significantly different, as the values themselves are already very different (Fig. 5), we examined inter-revolution difference to evaluate the consistency of each parameter (speed and duration) throughout the performance for each subject. Both values were found to be significantly different among groups (duration difference: Chi sq(2) = 19, p < 0.001; speed difference: Chi sq(2) = 18.7, p < 0.001) (Fig. 6A,B). Specifically, the mean inter-revolution difference for duration were low for the DP group compared to the SP (0.137 ± 2.134 ms and 10.88 ± 5.7 ms, respectively, p < 0.001); the mean inter-revolution difference for speed presented the opposite trend, with larger differences for DP group compared to the SP group (2.081 ± 0.413 mm/s and 0.531 ± 0.473 mm/s, respectively, p = 0.008). Inter-revolution differences for the NP group presented intermediate values (5.6 ± 4 ms for duration and 0.67 ± 1.24 mm/s for speed). Significant differences between the NP group and the DP were found for both duration and speed (p = 0.008, and p < 0.001, respectively), whereas no significant differences were found between the NP and the SP groups for duration and speed (p = 0.06, and p = 0.99, respectively).

To broaden our examination of the potential differences between the groups, the number of peaks within the speed profiles for each group were evaluated revealing that a smoother performance is present for the SP (124.8 ± 7.2 average peaks) regarding speed compared to the DP group (145.8 ± 18.4), also in this case the NP group presented intermediate values (131.7 ± 13.1). Significant differences were found among groups (Chi sq(2) = 7.1, p = 0.02). Specifically, between DP and SP groups (p = 0.02) but not between DP and NP group (p = 0.17) or between the SP and NP groups (p = 0.44).

**Figure 7:**
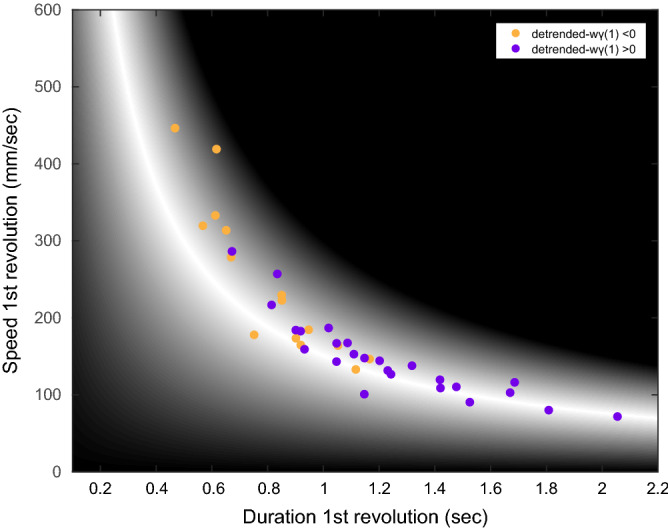
Detrended windowed lag(1)-autocorrelation results. Scatter plot of the detrended windowed lag(1)-autocorrelation values, every dot represents a subject and is colored according to the lag(1)-autocorrelation value with negative values in yellow and positive in purple. It is possible to note that the more subjects are found near the extreme, they tend to have either positive values (for speed preference) or negative values (for duration preference).

In addition, we implemented a timing variability analysis using a detrended windowed lag(1)-autocorrelation (detrended-wγ(1)) on the duration values of the 3 groups (Fig. 7). For the DP group (with the exception of 2 subjects) values were negative (− 0.138 ± 0.255), suggesting that the group as a whole employs a more event-based type of control. For the SP group, values were invariably positive (0.290 ± 0.211), suggestive for a more emergent-based type of control. The NP group represented the intermediate case with both positive and negative values (with 9 subjects showing negative values, group average 0.105 ± 0.223). Finally, significant differences were found among groups (Chi sq(2) = 10.45, p = 0.005). Post hoc analysis revealed that the differences among the groups were mainly due to the differences between the SP and the DP groups (p = 0.003), whereas no significant differences were found for other comparisons (p = 0.07 for DP vs NP, p = 0.26 for SP vs NP).

**Figure 8:**
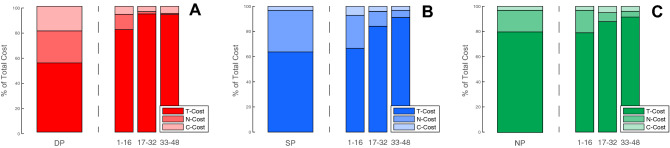
TNC-cost relative weight. The bar plot represents the relative weight of each of the cost components for each group. It is possible to see that for the overall performance, the weight of Noise is very large for the SP group (**B**, left side) and Covariance is negligible, whereas for the DP group (**A**, left side) both noise and covariance has a relatively equal weight. When investigating the segmented performances, it is possible to see that throughout the performance for the DP group (**A**, right side) noise reduces very quickly, accounting for small weight already in the middle of the performance, whereas for the SP (**B**, right side) it tends to reduce more gradually. It is also possible to note that the relative weight of tolerance was very high for the NP group for the overall performance (**C**, left side) as well as throughout the performance (**C**, right side).

**Figure 9:**
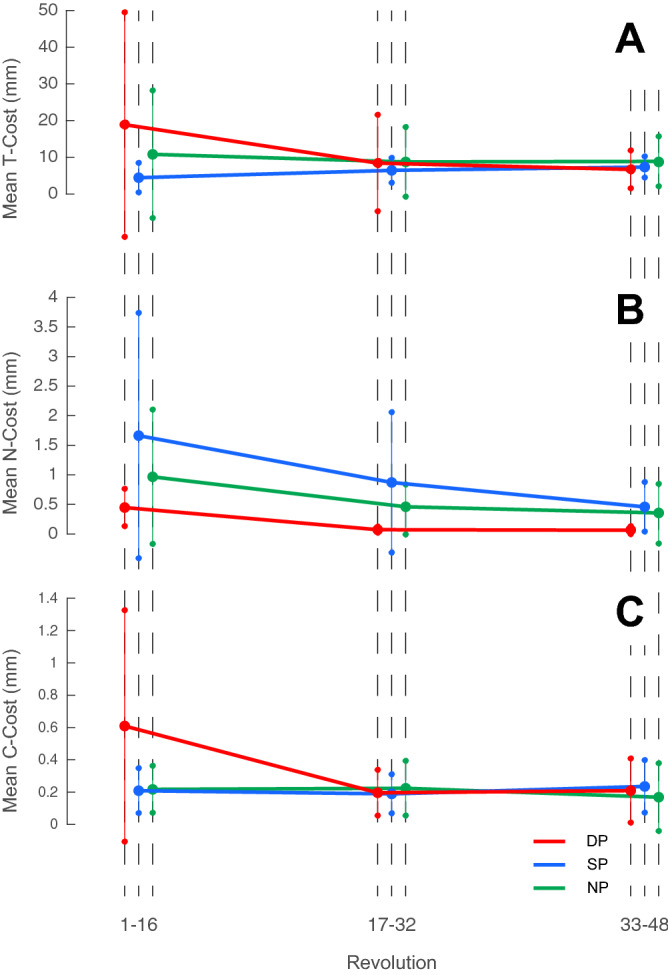
TNC-cost throughout performance. Results for the tolerance (**A**), noise (**B**), and covariance (**C**) costs throughout the performances for each group. While for the DP group it is possible to see that all costs reduce throughout the performance, for the SP group, only noise shows a very big reduction is visible whereas the tolerance and covariance remain relatively stationary.

To better understand whether or not strategies tend to change during the performances we implemented a Tolerance, Noise and Covariance analysis (TNC). The TNC-cost analysis was first implemented on the complete performances. Following that, in order to get a clearer image of the performance as it progresses, we divided the data into to 3 segments, initial (16 revolutions), middle (16 revolutions) and end (16 revolutions), and re-implemented TNC analysis (Figs. 8 and 9; Table 2).

**Table 2:** TNC-cost analysis results.

	Duration preference			Speed preference			No preference		
	T	N	C	T	N	C	T	N	C
Initial	81 ± 17% (18.9 ± 30.6 mm)	12 ± 17% (0.44 ± 0.31 mm)	6.3 ± 11% (0.6 ± 0.71 mm)	66 ± 22% (4.4 ± 4 mm)	26 ± 23% (1.6 ± 2 mm)	7 ± 9% (0.2 ± 0.13 mm)	82 ± 22% (10.8 ± 17.3 mm)	13 ± 22% (0.9 ± 1.1 mm)	4.2 ± 2.4% (0.21 ± 0.14 mm)
Middle	93 ± 3% (8.4 ± 13.1 mm)	1.7 ± 1.2% (0.07 ± 0.05 mm)	4.2 ± 3.7% (0.19 ± 0.14 mm)	84 ± 14% (6.4 ± 3.3 mm)	11 ± 15% (0.87 ± 1.1 mm)	4 ± 5.3% (0.18 ± 0.12 mm)	87 ± 12% (8.7 ± 9.4 mm)	7 ± 8.9% (0.38 ± 0.44 mm)	5 ± 6.9% (0.22 ± 0.16 mm)
End	93 ± 8% (6.6 ± 5.1 mm)	0.9 ± 0.7% (0.06 ± 0.06 mm)	5.5 ± 7.8% (0.2 ± 0.19 mm)	91 ± 5% (7.3 ± 2.8 mm)	5.6 ± 4.5% (0.45 ± 0.41 mm)	3.1 ± 2% (0.23 ± 0.16 mm)	91 ± 8% (8.8 ± 6.8 mm)	4.6 ± 5.8% (0.34 ± 0.5 mm)	3.8 ± 6.6% (0.16 ± 0.2 mm)
Overall	55 ± 35% (3.36 ± 5.2 mm)	25 ± 31% (0.31 ± 0.23 mm)	19 ± 25% (0.41 ± 0.46 mm)	63 ± 22% (4.4 ± 2.2 mm)	33 ± 22% (2.37 ± 1.56 mm)	3.1 ± 2% (0.2 ± 0.12 mm)	79 ± 18% (9 ± 9.8 mm)	17 ± 17% (1 ± 0.8 mm)	3.2 ± 4.5% (0.2 ± 0.22 mm)

In the table are reported the results obtained from the TNC-Cost analysis for each group (Duration Preference, Speed Preference, and No-Preference). Results for the individual costs are reported in the relevant columns (T = Tolerance, N = Noise, and C = Covariance) and are expressed as relative weight for each cost (in percentage) as well as the measured cost in mm (in parenthesis). Each row corresponds to the relative segment of the performance (initial, middle, and end), each composed of 16 revolution. The last row reports values of the overall performance (i.e., 48 revolutions).

It is possible to note that for all groups the major cost component was represented by the T-Cost. This is especially evident when considering the percentage of the cost of the overall performance, in which tolerance was responsible for 79% of the all cost for the NP group, compared to 55% and 63% for the DP and SP groups (respectively). When reviewing the segmented data, it is evident that Tolerance continued to play an important factor throughout the performance (Figs. 8 and 9). On the other hand, changes of the N-Cost, which invariably reduced throughout the performances for all groups (Fig. 9), could account for the improvement of the performances.

For the SP group, improvements seem to be mostly due to optimizing dispersion rather than exploration, as T-Cost values in the segmented analysis increase. That being said, T-Cost still represented the major contributor for the overall cost for the SP group, suggesting that exploration was lacking. For the DP group, while all costs reduced with the progression of the performance, the major component remained the T-Cost. For the NP group, T-Cost remained relatively stable, suggesting very little exploration. Improvements in this group appear to be sustained mostly by optimizing dispersion.

Significant differences among groups were found for T-Cost (Chi sq(2) = 7.3, p = 0.02) as well as for the N-Cost (Chi sq(2) = 12.9, p = 0.001). Specifically, for the T-Cost, significant differences were found between DP and NP (p = 0.02), whereas other comparisons did not reveal significant differences (DP vs SP p = 0.48, SP vs NP p = 0.54). For N-Cost, significant differences were found between DP and SP (p = 0.001), but not for other comparisons (DP vs NP p = 0.1, SP vs NP p = 0.07). No significant differences among groups were found for C-Cost (Chi sq(2) = 0.24, p = 0.8). Finally, for the segmented results, significant differences were found among groups only for the N-Cost at the end segment (Chi sq(2) = 10.8, p = 0.004). Specifically, post hoc analysis revealed significant differences between DP and SP (p = 0.004) and between DP and NP (p = 0.02), but not between SP and NP groups (p = 0.5).

## Discussion

In this paper we have shown that specific execution strategies may be identified in a continuous task and that strategies remain relatively stable throughout the performance (Figs. 5, 6). The identified strategies were those of maintaining a relatively constant speed or a relatively constant duration. Moreover, as the strategies remained relatively stable, the initial location of the performances in the execution space can be used to determine the subjects’ strategies. Finally, we demonstrated that, given the continuous nature of the task, exploration of the execution space was limited.

The two strategies identified in this study (i.e., maintaining speed or maintaining duration) could suggest different types of control. While duration is defined by a periodic measure (i.e., interval between repetition) and is best maintained when controlled periodically^29,30^, speed on the other hand is a more dynamic measure which is controlled continuously. This view may suggest that the SP group utilizes a more active type of control whereas the DP group a more intermittent one. In fact, examination of the peak number within the speed profile of the performances revealed a lower number of peaks for the SP compared to the DP, suggesting a smoother performance^21,22^. Also, the positive detrended-wγ(1) values for the SP further support the notion of a more dynamic type control, compared to negative values for the DP group which are suggestive for a more discrete type of control^31^. This is an interesting finding as it suggests that some people take a discrete approach and some a continuous approach for the same motor problem, specifically in this study, some subjects appear to take a discrete strategy in a continuous task. It was previously shown that continuous tasks, when performed intermittently, could present a more discrete type of control^32^. Conversely, discrete tasks could transition to continuous rhythmic movements under certain constraints^33^. However, the findings presented in this study demonstrate that even when un-provoked by any specific constraints which could favor a certain strategy, subjects still do diverge into different modes of control.

In terms of performance optimization, it is interesting to note that, differently to what was shown in other studies^9,28^, results here suggest that subjects tend not to sufficiently explore the execution space. As demonstrated by Cohen and Sternad 2009, tolerance cost was the first one to reduce during a performance, reaching values that are near zero relatively quickly, thus leaving noise and covariance as the determining factors for optimization as performances progress. In our study, although an initial reduction was found for tolerance in all groups, it still invariably represented the main component of the overall cost. If subjects indeed tended to change strategies during the performances, a greater reduction in T-Cost would have been expected. Therefore, taking together the inter-revolution data (Fig. 8), the detrended-wγ(1) (Fig. 7), as well as the apparent lack of exploration, results seem to suggest that subjects do not change their strategy during the performance. This is more evident for subjects in the NP group, who already at the beginning of the performance were located at the area of greater tolerance and, as such, demonstrated the least change in tolerance cost (i.e., least exploration).

The premise of this study, of emergence and identification of strategies during a performance, is similar to a previously published study. King and colleagues, 2012^9^, examined exploitation of different strategies in a star tracing task with the goal of minimizing a performance score that was given as feedback. Specifically, 3 clusters of individuals, corresponding to different search strategies (i.e., maintaining speed, maintaining accuracy, and a mixed strategy), were identified from the initial performance. The study reported that differences between the groups also persisted during the performance, and were reflected as a differential modification of spatial and temporal components throughout the performances between the groups.

While the general premise of the study goes in line with our findings, (i.e., separation of subjects, persistence of strategies), some key differences should be noted. King’s study employed a more discrete approach, in which the tracings of the shape were performed one tracing at a time, presenting a score to the participants upon completion of each trial. The interval between trials could allow for elaborations and corrections for the following trial, which would lead to greater exploration and possibly also strategy changes. In fact, the researchers reported a high exploration index for all groups, and the mixed group included in the study was composed of subjects that took advantage of both strategies. In this current study, we employed a continuous approach, in which the subjects did not stop to assess their performance, thus minimizing possible elaborations and making it less likely to introduce abrupt changes in strategy. In fact, the results in this study suggest that exploration is insufficient across groups and also that subjects do not seem to shift from one strategy to another.

The discrepancies between the results of the two studies are most likely due to the difference in the nature of the task (discrete vs continuous). As stated earlier, in continuous tasks, pre-movement planning is less important^19,20^, which could translate into small incremental changes in performance and, therefore, could account for the reduced exploration. Another possible explanation for the discrepancy in exploration between King’s study and this study, is that subjects received feedback regarding their performance and knew, prior to the task, that improvements of their score could be obtained by means of modifying one of the variables (speed or accuracy). On the other hand, in our study, even though subjects received a continuous visual feedback regarding their performance, knowledge on improvement was only implicit, as the given instructions only indicated to trace as fast and as accurate as possible.

We should also note that in order to examine exploration in this study, we used the TNC-Cost analysis instead of the Exploration Index used by King and colleagues. The exploration index was based on a cumulative measure of the distance travelled within the space across trial, normalized by the maximum Euclidean distance between any two trials. While this is an appropriate measure when performances are dispersed within the execution space, we believe that for clustered performances (i.e., performances in which all trials are relatively centered around one point), this type of analysis could cause an over-estimation of the exploration, providing more an estimation of exploration within the cluster, rather than the entire execution space, which could also account for the discrepancy between the two studies.

A few limitations of this study should be noted. The first, it is important to note that while the execution variables in this task demonstrate differences, these variables are chosen and are a small example of many. In this study since we have focused on the distance measure as the result variable, the choice of the execution variables (i.e., speed and duration) was based on their ability to quantify the result variable. However, by choosing different variables there is also the possibility for other patterns to emerge. Since the goal of this paper was limited to the presentation of an approach for identifying strategies from the initial conditions, the examination of other execution variables, though highly encouraged for future studies, is beyond the scope of this study. Another limitation is related to the result variable in this study (i.e., distance), which was used as a surrogate of the radius variable. Since the result variable used was not explicitly part of the instructions (i.e., perform as fast and as accurate as possible), part of the differences observed could be due to different interpretations of the task by the participants. We believe however that, as there is a high correlation between the result variable and the radius (which is considered as a measure for accuracy), as well as the gradual reduction in radius error observed (Fig. 4), the possibility of different interpretations is minimal.

Given the short time span of each trial (averaging 42.3 ± 12.1 s across all subjects), the role of learning in the current study is debatable. Although some motor adaptation studies have described that adaptation could also occur in a relatively short time span^20,34,35^, the role of learning in this current study was not investigated as the scope was that of determining the existence and predictability specific strategies during the performance of a continuous task. Therefore, though we cannot confirm the specific contribution of learning in accounting for the observed results, we cannot fully exclude it either. It should also be considered that though initial strategies used do not change appreciably in this study, it may be due to the short time interval of the execution. Future studies would benefit also examining strategy modifications over different time scales.

Also, it should be considered that performances are likely to vary greatly depending on the task as well as the task requirements. This study was concentrated on a continuous task, and the results reported present differences compared to those obtained by a discrete task^9^. Though a side-by-side comparison of the two was not included in this study, it would be interesting to examine whether subjects maintain performance patterns also across tasks. Finally, though this study focused on a group division of the subjects, this division was based on the observation that subjects at the extremes of the spectrum possess different characteristics in terms of performance. The small sample size following the group division renders it difficult to draw definitive conclusions, and future studies would benefit from increasing the sample size. Still, the decision for group separation was made in order to evidence and quantify these differences, however, we believe that the trend shown in the study is not as net as presented but much more gradual and would be better viewed as a continuum.

There is a plethora of possible explanations as to why a performance may occur in a certain way. In this study we focused mostly on the existence and modification of execution strategies. However, since these strategies are determined already at the beginning of the performance, it is possible that subjects present certain biases/inclination toward a certain strategy, which would then after determine their initial location within the execution space. It is known that while the possibilities for movement execution are infinite, most subjects tend to demonstrate certain stereotypical patterns^36^. In fact, previous work on exploration strategies demonstrated that individuals approach differently motor problems in a way that is dependent on the subject’s perception and action repertoire^10^, and that strategies remain consistent across various manipulations and are dependent on individual preferences^9,10,12^. These individual preferences were also found in reaching as directional biases^37^ as well as differences in feedback reliance among individuals^38^. Therefore, it is also possible that subjects possess some estimation of their own control, and consequently, by being aware of their own limitations would be more inclined toward one direction or the other. Though it is debatable whether the results provided in this study are indeed a manifestation of personal biases, it would be interesting in future studies to examine how knowledge of these possible biases/inclinations by the subjects may affect their performance in successive trials.

## Conclusions

The ability to determine how a task will be performed may help subjects become more aware of certain aspects of their performances, allowing them to revise their execution strategies, as well as make them aware of certain execution patterns they were not previously aware of, thus allowing them to achieve better performances. Though there are different factors that were suggested to influence performances, by focusing solely on success as an outcome measure, their predictive power may be limited. Here we shifted the focus away from “how well a task is performed”, and investigated the differences in “how the task is performed” between individuals. Indeed, we identified two specific execution strategies in the given task suggestive for engagement of different types of control. We believe that by examining the strategies employed, it could be easier to understand which aspects of performances could be improved and individual differences could be better investigated.

## Supplementary Information

Supplementary Information
