# Supplementary material for: Examining modifications of execution strategies during a continuous task
Source: Sci Rep. 2021 Mar 1;11:4829. doi: 10.1038/s41598-021-84369-5 (PMC7921105; doi:10.1038/s41598-021-84369-5)
Supplement: Supplementary file 1 — Supplementary Information [file 41598_2021_84369_MOESM1_ESM.pdf]

Examining modifications of execution strategies during a continuous task

Erez James Cohen[1], Kunlin Wei[2], Diego Minciacchi[1]

[1]Department of Experimental and Clinical Medicine, Physiological Sciences Section, University of Florence, Florence, Italy

[2]School of Psychological and Cognitive Sciences and Beijing Key Laboratory of Behavior and Mental Health, Peking University, Beijing, China.

Corresponding author: Diego Minciacchi,

Department of Experimental and Clinical Medicine, Physiological Sciences Section, University of Florence, Viale Morgagni 63, I-50134 Florence, Italy

E-mail address: diego.minciacchi@unifi.it.

Supplementary Material

In this supplementary material section, we present the results obtained from the division of the dataset into 2 groups instead of 3 groups. Subjects were therefore divided into 2 groups using a k-means algorithm, using only the speed and duration of the first revolution (Figure S1A). To apply the k-means algorithm, a priori knowledge regarding the quantity of groups is needed, this was derived from our theorized division of the solution manifold into two parts. The 2 groups obtained were that of steady speed (i.e., Speed Preference, SP; n=21) and steady duration (i.e., Duration Preference, DP; n=19). Particularly, the average duration measured 0.703±0.16 sec for the DP group, 1.01±0.25 sec for the SP group. The average speed measured 230±6.2 mm/sec for the DP group, 155±9.1 for the SP group. Both duration and speed were found to be significantly different between groups (for duration Chi sq(1)=71.2, p<.001; for speed, Chi sq(1)=71.2, p<.001).

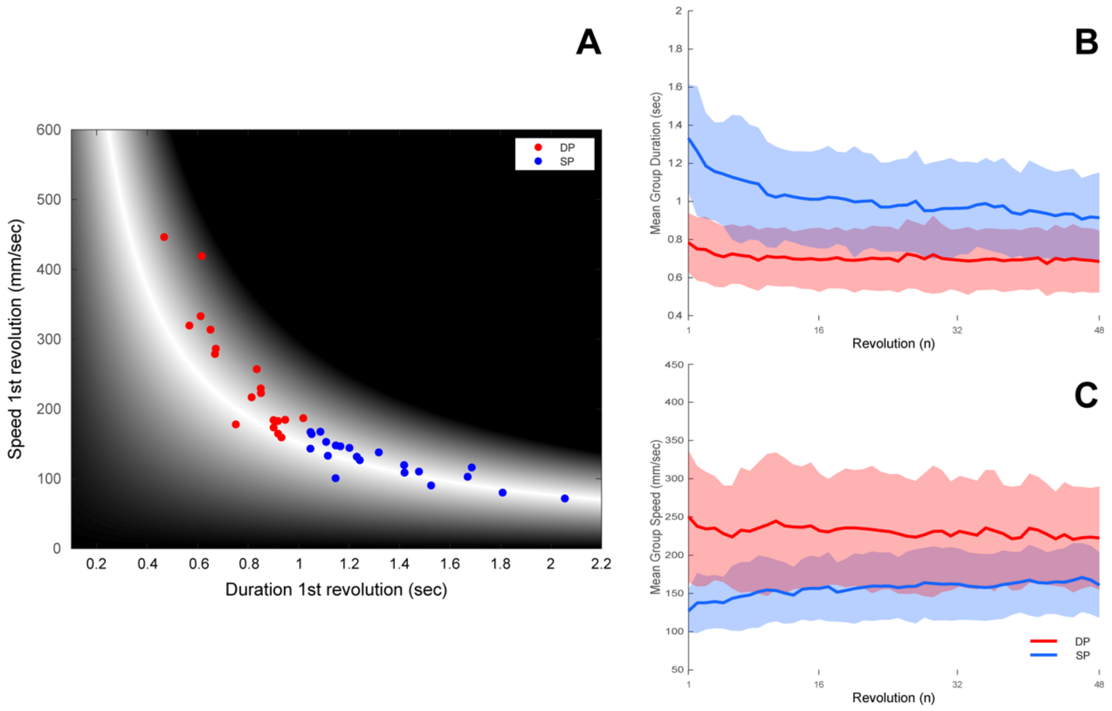

Figure S1. Group Division. Subjects were subdivided into 2 groups following the implementation of k-means on the speed and duration values of the first revolution. **A.** Scatter plot of the speed and duration values obtained on the first revolution, every dot represents a subject and is colored according to the k-means division into a Duration Preference group (i.e., DP; n=19; red), Speed Preference group (i.e., SP; n=21; blue).

B. Duration results throughout the performance for the 2 groups. It is possible to note that the DP group (red) remains relatively stable compared to the SP group (blue). Specifically, the DP group mean duration measured 0.78±0.15 sec for the first revolution, and remained relatively stable throughout the performance reaching 0.68±0.16 sec by the last revolution. The SP group measured 1.33±0.28 sec for the first revolution, reaching 0.91±0.23 sec by the last revolution. **C.** Speed results throughout the performance for the 2 groups, demonstrating that SP group (blue) remains relatively stable compared to the DP group (red). Specifically, the DP group averaged 249.2±85.3 mm/sec for the first revolution, reaching 221.4±66.9 for the last revolution. The SP group averaged 126.7±27.7 mm/sec for the first revolution and reaching 160.3±42.3 mm/sec by the last revolution.

If strategies are indeed fixed, there should be a minimal change in duration for the DP group along with large changes in duration for the SP group (Figure S1B). The opposite should be visible when examining the speed parameter (Figure S1C). Consequently, performances would be accommodated by a greater modification of a single execution variable. Since using the speed and duration values would evidently be significantly different, as the values themselves are already very different (Figure S1), we examined inter-revolution difference to evaluate the consistency of each parameter (speed and duration) throughout the performance for each subject. Both values were found to be significantly different between the groups (Figure S2A, S2B). Specifically, the mean inter-revolution difference for duration were low for the DP group compared to the SP (3.2±2.6 msec and 8.9±4.8 msec, respectively, Chi sq(1)=17.2, $p<.001$); the mean inter-revolution difference for speed presented the opposite trend, with larger differences for DP group compared to the SP group (1.47±1.03 mm/sec and 0.74±0.79 mm/sec, respectively, Chi sq(1)=6.01, $p=.01$).

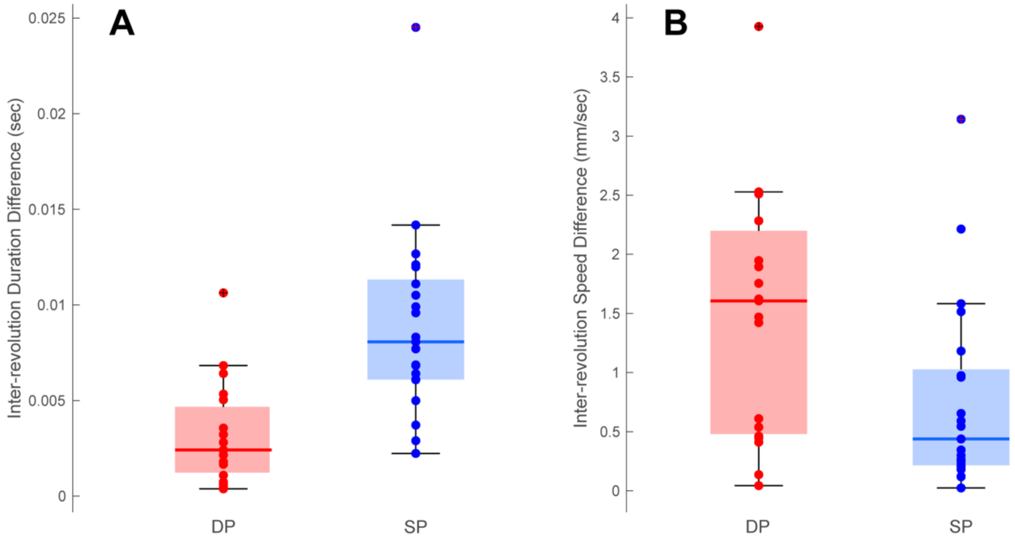

Figure S2. Inter-Revolution Differences. Box plot representing the group inter-revolution differences for duration (**A**) and for speed (**B**). It is possible to see that, for duration, DP group (red) presents very small differences compared to SP (blue), suggesting that DP group maintains the duration relatively constant. The opposite trend is visible for speed in which the SP group (blue) presents smaller differences compared to the DP group (red). The colored dots superimposed on the plots represent the individual subject and are color coded according to their group (red for DP group, and blue for the SP group). Red crosses represent extreme outliers.

The number of peaks within the speed profiles for each group were evaluated revealing that a smoother performance is present for the SP (130±10.1 average peaks) regarding speed compared to the DP group (136.3±18.3). Though no significant differences were found between the groups (Chi sq(1)=1.5, p=.2). The SP group tended to be slower compared to the DP group (49.4±11.3 sec for SP compared to 34.5±7.4 for the DP group, Chi sq(1)=12.6, p<.001).

Timing variability analysis was conducted using a detrended windowed lag(1)-autocorrelation (detrended-wγ(1)) on the duration values of the 2 groups. For the DP group (with the exception of 7 subjects) values were negative (-

0.062±0.22), suggesting that the group as a whole is employs a more event-based type of control, driven by an internal representation of time. For the SP group (with the exception of 3 subjects) values were positive (0.236±0.214), suggestive for a more emergent-based type of control, driven by movement dynamics. Finally, the two groups were found to be significantly different from each-other (Chi sq(1)=12.3, $p<.001$). Even though there is not a net division between the groups for the lag one values, inspection of the figure does suggest that negative lag values predominate on the DP area, the opposite is true for the SP area.
